# Impact of Grade Groups on Prostate Cancer-Specific and Other-Cause Mortality: Competing Risk Analysis from a Large Single Institution Series

**DOI:** 10.3390/cancers13081963

**Published:** 2021-04-19

**Authors:** Daimantas Milonas, Tomas Ruzgas, Zilvinas Venclovas, Mindaugas Jievaltas, Steven Joniau

**Affiliations:** 1 Medical Academy, Department of Urology, Lithuanian University of Health Sciences, 44307 Kaunas, Lithuania; zilvinas.venclovas@gmail.com (Z.V.); mindaugas.jievaltas@lsmuni.lt (M.J.); 2 Department of Urology, University Hospital of Leuven, 3000 Leuven, Belgium; steven.joniau@uzleuven.be; 3 Department of Applied Mathematics, Kaunas University of Technology, 44249 Kaunas, Lithuania; tomas.ruzgas@ktu.lt

**Keywords:** prostate cancer, radical prostatectomy, grade groups, cancer-specific mortality, other-cause mortality

## Abstract

**Simple Summary:**

For prostate cancer patient, information on risk of long-term cancer-specific and other-cause mortality is essential to avoid over- and undertreatment. Patient stratification to low-, intermediate-, and high-risk groups has been used for decades. However, recent evidence has shown that such stratification is not optimal and outcomes differ widely, especially in high-risk prostate cancer patients. Gleason score grading is an important factor for the prediction of cancer-specific survival and has been included in all prostate cancer risk stratification models. Moreover, this parameter could be used as an independent predictor. Recently proposed grade group model demonstrated good predictive probability on short-term outcomes. However, there is a lack of data regarding long-term cancer-specific survival. In the presented study, we analyzed long-term oncological outcomes in different grade groups. Detected ratio between cancer-specific and other-cause mortality could be very informative and helpful in prostate cancer patient risk stratification and more precise clinical decision making.

**Abstract:**

*Objective:* To assess the risk of cancer-specific mortality (CSM) and other-cause mortality (OCM) using post-operative International Society of Urological Pathology Grade Group (GG) model in patients after radical prostatectomy (RP). *Patients and Methods:* Overall 1921 consecutive men who underwent RP during 2001 to 2017 in a single tertiary center were included in the study. Multivariate competing risk regression analysis was used to identify significant predictors and quantify cumulative incidence of CSM and OCM. Time-depending area under the curve (AUC) depicted the performance of GG model on prediction of CSM. *Results:* Over a median follow-up of 7.9-year (IQR 4.4-11.7) after RP, 235 (12.2%) deaths were registered, and 52 (2.7%) of them were related to PCa. GG model showed high and stable performance (time-dependent AUC 0.88) on prediction of CSM. Cumulative 10-year CSM in GGs 1 to 5 was 0.9%, 2.3%, 7.6%, 14.7%, and 48.6%, respectively; 10-year OCM in GGs was 15.5%, 16.1%, 12.6%, 17.7% and 6.5%, respectively. The ratio between 10-year CSM/OCM in GGs 1 to 5 was 1:17, 1:7, 1:2, 1:1, and 7:1, respectively. *Conclusions:* Cancer-specific and other-cause mortality differed widely between GGs. Presented findings could aid in personalized clinical decision making for active treatment.

## 1. Introduction

Clinical decision making in prostate cancer (PCa) patients is very complex and information on risk of long-term cancer specific mortality (CSM) and other-cause mortality (OCM) is essential to avoid over- and undertreatment. Previous reports from population-based or multi-institutional large series provide such information [1,2,3,4]. In these studies, patients were stratified in low-, intermediate-, and high-risk group according to the proposed risk group models based on preoperative prostate specific antigen (PSA), stage, and Gleason score [5,6]. However, recent evidence has shown that such stratification is not optimal and outcomes differ widely, especially in high-risk PCa patients [7,8,9]. Gleason score grading is an important factor for the prediction of cancer-specific survival and has been included in all PCa risk stratification models. Moreover, this parameter could be used as an independent predictor. Reports, especially those after 2014 International Society of Urological Pathology (ISUP) recommendations [10], demonstrate the growing role of Gleason score in prognosis of oncological outcomes. The new ISUP Grade Group (GG) model shows better performance in comparison with former Gleason grading both on prediction of biochemical progression-free survival [11,12] and the forecast of cancer-specific survival [13,14] and is now accepted and widely used [9]. Indeed, to transfer information of cancer-specific and overall survival from different reports to individual patient’s risk of dying from cancer or other causes is complicated. One of the most realistic ways to receive reliable information on mortality is competing risk analysis. Taking into consideration that Gleason grading is a key predictor of CSM, such data would be very useful and friendly to use for both clinicians and patients in order to choose the optimal personalized treatment option. 

We hypothesized that GG model could be an important predictor of CSM and provide correct information on risk of death from cancer and other causes in long-term follow-up. Additionally, we focused on patient age and stratified men into two groups aged <65 and ≥65 year. For these purposes, competing risk analysis was chosen in a large series of patients who underwent radical prostatectomy (RP) in a single tertiary center.

## 2. Patients and Methods

**Figure 1:**
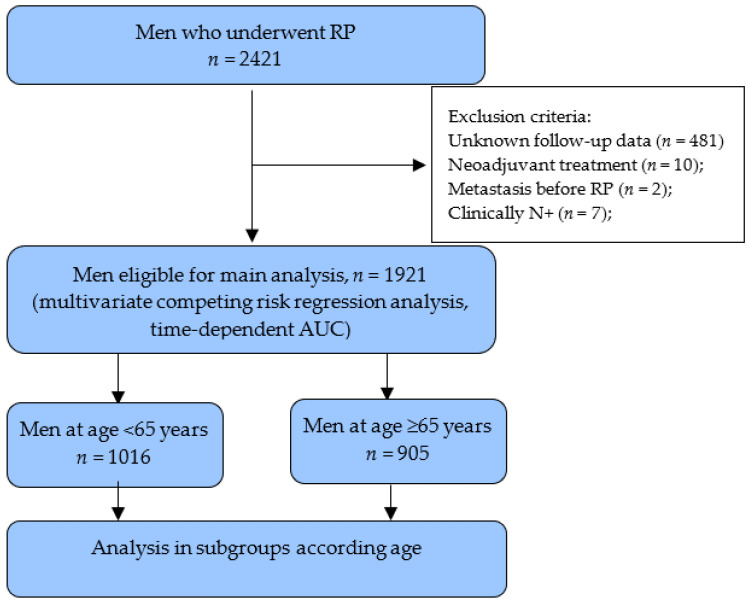
Study flowchart. RP—radical prostatectomy, AUC—area under the curve.

Between 2001 and 2017, 2421 men were treated by RP for clinically localized PCa at a Lithuanian University of Health Sciences Department of Urology. All patients’ data were included in a PCa database. Exclusion criteria consisted of neoadjuvant treatment, unknown follow-up data, M+ disease, and clinically N+ (Figure 1). Clinical characteristics, such as age, preoperative PSA, clinical stage (cT), and biopsy GGs were collected before RP. Pathological parameters (stage (pT) and GGs, lymph nodes (pN), and surgical margin (SM) status) were registered after RP. Pathological stage was assessed using 2002 TNM system, and tumor grading was classified using revised 2005 ISUP Gleason score grading system and the suggested new GG model after 2014 [15,16]. The local ethical committee approved the collection and analysis of the data (Nr. BE-2-48).

Primary endpoints were cancer-related deaths (CRD) and deaths from all causes. Data about patient death were taken from national health care database. All cases of death were rechecked with follow-up data available in the center database. Death without clinically proved PCa progression was assigned as other-cause death. Time to CRD and other-cause death was defined as the time interval from RP to the event. 

Frequencies and proportions or medians with interquartile ranges (IQR) were reported for categorical or continuous variables, respectively. Baseline characteristics between younger and older men were assessed using Chi-square for categorical data and Mann–Whitney test for continuous data. The Fine and Gray model was used for data analysis accounting for death of other causes as a competing risk. A multivariate competing risk regression analysis was used to identify predictors of CSM. We estimated 10-year cumulative incidence accounting for the competing risk of death from other causes according to GG model in all cohort. Furthermore, we stratified patients by age into groups <65 and ≥65 years. We quantified cumulative incidence of CSM and OCM in different age groups using GG model. Finally, the discriminative performance of the GG model was quantified by time-dependent AUC that demonstrated variation of predictive probability of the model at each follow-up year. Results were presented as hazard ratios (HR) with 95% confidence interval (CI). Analyses have been performed using SAS software (version 9.4 of the SAS System for Windows) with two-sided significance level set at *p* < 0.05. 

## 3. Results 

Median follow-up after RP for survivors was 7.9 year (IQR 4.4–11.7). Of the 1921 patients in our series, 235 (12.2%) died of other causes and 52 (2.7%) died of PCa. Patient characteristics are represented in Table 1. Overall, 5- and 10-year CSM was 1.2% (95% CI 0.8–1.9) and 4.1% (95% CI 3.1–5.6), respectively. The 5- and 10-year OCM was 5% (95% CI 4.1–6.2) and 15.5% (95% CI 13.6–17.7), respectively. 

**Figure 2:**
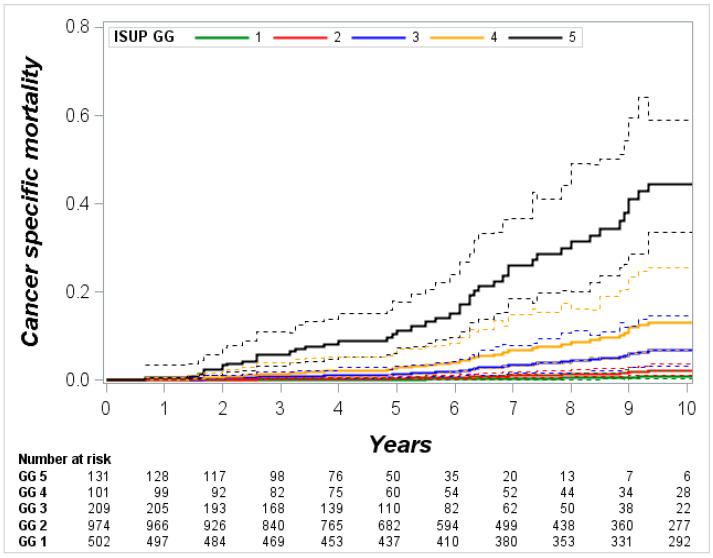
Cumulative risk of cancer-specific mortality in postoperative International Society of Urological Pathology Grade Group (ISUP GG) model.

Multivariate competing risk regression analysis established significant predictors of CSM. The strongest of them was GG (*p* < 0.0001), followed by age (*p* = 0.001), lymph node invasion (*p* = 0.003), stage (*p* = 0.01), and SM status (*p* = 0.02) (Table 2). The risk of CSM increased by each GGs, and in GG5, this risk was 20-fold higher (HR 20.3, *p* < 0.0001, Figure 2) comparing with GG1. However, GG2 was not at a significantly higher risk of CSM in comparison with GG1 (HR 1.9, *p* = 0.3), as well as GG3 compared to GG2 (*p* = 0.07) and GG4 compared to GG3 (*p* = 0.5).

**Figure 3:**
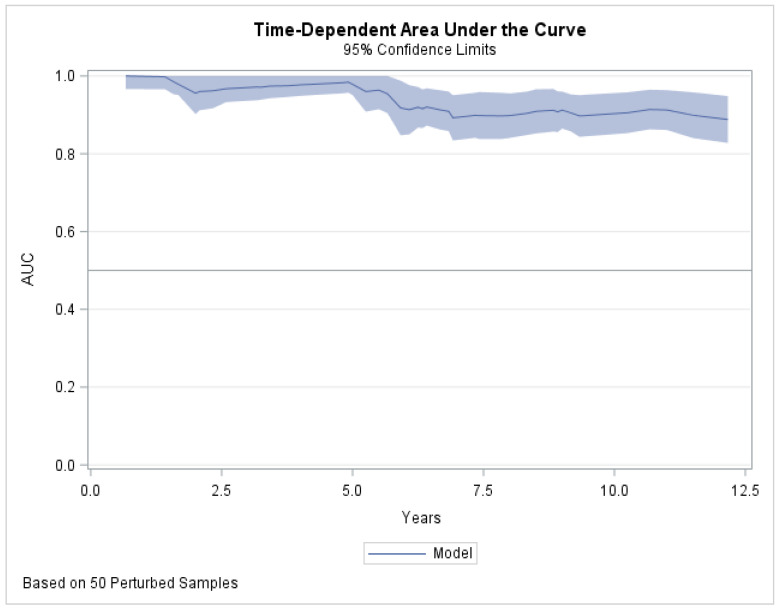
Time-dependent area under the curve (AUC) depicted prediction of cancer-specific mortality for postoperative International Society of Urological Pathology Grade Group model at each follow-up year.

GG, based on pathological findings, showed very high and stable (time-depending AUC 0.88, Figure 3) performance for prediction of CSM.

**Figure 4:**
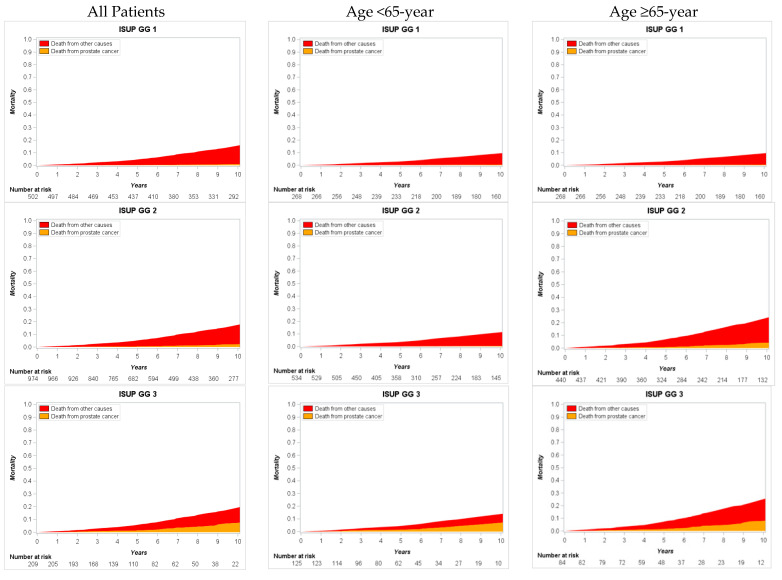

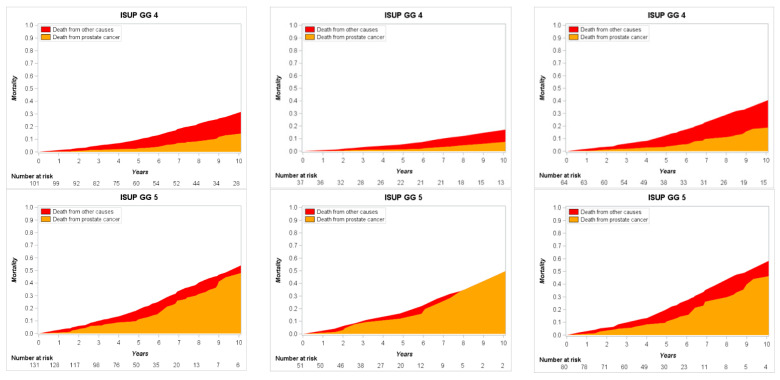
Cumulative cancer-specific and other-cause mortality in postoperative International Society of Urological Pathology Grade Group (ISUP GG) model stratified by patient age.

Cumulative 10-year CSM increased gradually from 0.9% (95% CI 0.3–2.2) in GG1 to 48.6% (95% CI 33.9–69.7) in GG5; cumulative 10-year OCM in GGs 1–4 was similar and varied from 12.6% (95% CI 7.9–22.9) to 17.7% (95% CI 12.6–32.7), while in GG5 OCM reached only 6.5% (95% CI 2.6–21.2) (Figure 4, Table 3). Assessing proportion between death of cancer and death of other causes at 10-year after RP showed wide differences between GGs. The ratio of CSM/OCM was 1:17 in GG1, 1:7 in GG2, 1:2 GG3, 1:1 in GG4, and 7:1 in GG5 (Table 4). 

Furthermore, we stratified patients according to age into <65- and ≥65-year and focused on cumulative mortality in these subsets. Naturally, older men demonstrated increased 10-year OCM compared with younger men: 21.3% (95%CI 18.1–25.2) vs. 10% (95%CI 7.7–12.9), respectively. Importantly, in older counterpart 10-year CSM was up to 3-fold higher compared to younger men—6.1% (95% CI 4.3–8.5) vs. 2.3% (95% CI 1.3–4.0), respectively. 

In younger men subset, cumulative 10-year CSM was similar in GG1 and GG2 (0.4% and 0.3%, respectively), as well as in GG3 and GG4 (7.3% and 7.6%, respectively), while in GG5, cumulative 10-year CSM increased up to 50.7%; 10-year OCM in GGs 1 to 4 varied marginally from 7% to 11.3%. No patients at 10-year died from other causes in younger counterpart (Figure 4, Table 3).

In older men subset, 10-year CSM increased by GGs 1 to 5 more gradually (1.4% vs. 4.3% vs. 8.1% vs. 19% vs. 46.7%, respectively). The lowest 12.8% 10-year OCM was in GG5, while in GGs, 1 to 4 mortality was similar and varied between 18.1% and 22.6% (Figure 4, Table 3).

The risk of death from PCa or other causes according to GGs at 10-year after RP is presented in Table 4. Younger men with GG1 and GG2 had the highest ratio (23:1 and 37:1, respectively) to die from some other cause than from PCa. Contrarily, in GG5 at 10-year follow-up, all patients died from PCa. Older counterpart demonstrated similar ratio of CSM/OCM as that detected in all cohort.

## 4. Discussion

PCa risk stratification is a rapidly evolving process, including increasing use of molecular and genomic tests and new molecular imaging techniques [17,18]. However, complexity of stratification models required information not always available in daily practice. Tumor grade definitely is one of the most important factors that forms the background for clinical decision making in PCa patients [6]. Long-term CSM and OCM outcomes using GGs could be a very informative and user-friendly tool in discussion with patients about prognosis of following treatment.

Our analyses confirm that GG is a robust predictor of CSM. The risk of CSM increased by each GGs, except GG2, in comparison with referent GG1, and in GG5, this risk increased 20-fold (HR 20.3, *p* < 0.0001). Moreover, GG model demonstrated very high performance (time-depending AUC 0.88) that was stable during all study period. These findings are in concordance with previous reports in which GG was identified as an independent predictor of CSM with high 0.75–0.82 discriminative probability [19,20,21].

Furthermore, we focused on cumulative CSM and OCM. Despite the number of studies analyzing GG impact on PCa progression at different endpoints, CSM data are scant. Indeed, for most authors the goal was prediction probability of GG rather than survival analysis. Pompe et al. reported 8-year CSM rate in RP subset from 0.5% and 0.9% in GGs 1 and 2 to 14.4% in GG5 [20]. He et al. presented the cumulative incidence of 7-year CSM following RP in GG1 0.43%, in GG2 0.48%, and in GG5 4.6% [22]. We noticed that cumulative 10-year CSM in GGs varies in a wide range: from 0.9% and 2.3% in GG1 and GG2 to 48.6% in GG5. Importantly, in GGs 1 and 2 the risk of 10-year OCM was 17- and 7-fold higher in comparison with CSM, and only in GG5, CSM risk was 7-fold bigger than OCM risk at 10-year after RP. Such findings provide insight that curative treatment for men with GG1, and probably for men with GG2, is at very high risk of overtreatment. Our results are in agreement with recent reports from trials that demonstrated no survival benefits for low risk PCa treated with RP vs. no treatment at very long-term follow-up [23,24]. On the other hand, men with GG5 represent an extremely poor prognosis subgroup and are candidates for very aggressive multimodal and probably novel treatment. 

Patient stratification according to age revealed several trends. Older counterparts had 3-fold higher risk of 10-year CSM in comparison with younger men, and this was especially true for GGs 1 to 4. We found that older men associated more often with GG4 and GG5, as well as with pT3b-4 disease, and this could be an explanation of higher CSM. These findings and our previous analyses [25] are in agreement with reports that demonstrated close association between older age and worse cancer features in a number of large population-based studies [1,26]. Contrarily, younger patient in GGs 1 and 2 demonstrated very low 10-year CSM of 0.4% and 0.3%, respectively, and very high risk of death from other causes (CSM/OCM ratio of 1:23 and 1:37, respectively). This could be a very important message in the discussion with younger PCa patients. However, younger counterparts demonstrated extremely poor outcomes in GG5: all patients at 10-year after RP died from PCa, and these men represent potentially lethal subgroup. 

The main challenge of present analyses is how our findings correlate with mortality results using other risk stratification models. Boehm et al. in 8741 patients after RP series demonstrated 0.2–0.6% of 10-year CSM in low, 2.3–4.4% in intermediate, and 14.8–19% in high CAPRA-S risk groups stratified by age [27]. Similar results from a Swedish nationwide population-based study in risk groups created by D’Amico criteria were reported by Van Hemelrijck et al. [4]. For patients undergoing RP, radiotherapy, or surveillance, 10-year CSM was 0.3–3% in low risk, 2–8% in intermediate, and 9–15% in high-risk group stratified by age and Charlson comorbidity index (CCI). Our findings are in agreement with the mentioned results comparing GG1 vs. low risk, GG2–3 vs. intermediate risk, and GG4–5 vs. high-risk. However, CSM in GG5 was up to 3-fold higher in comparison with GG4. The same significant survival difference between biopsy and postoperative GG4 and GG5 was presented by Ham et al. [28]. Heterogeneity of high-risk PCa group was reported by various studies [7,8,9,21], and probably a more strict definition is needed for this subset.

OCM at 10-year in our study was 15.5% and varied from 7% to 23%, except 0% in younger GG5 subgroup. These findings are higher than those in Boehm et al. study [27], but are in concordance with other previously reported studies [2,3,4] and demonstrate a similar age-dependent trend.

Taken together, our study findings demonstrated that GG model is the strongest independent predictor of CSM with high time-dependent performance. Cumulative 10-year CSM is in agreement with mortality rates assessed using other risk stratification models. Moreover, GG5 identifies the most aggressive PCa with probably lethal disease course. Presented CSM/OCM ratio represents leading cause of death among GGs. Finally, presented mortality curves are simple to use and could be very informative in discussing different risks after RP with each patient. They can also be helpful in personalized decision making to avoid over- and undertreatment of potentially indolent and aggressive PCa.

Our study is not devoid of limitation. We did not collect data on comorbidity in our series. However, studies demonstrated low comorbidity of men who underwent radical treatment [29]. Therefore, our detected 10-year OCM results in GGs could reflect real risk of death from other cause and could be used for the interpretation of mortality. In our study, specimen grade was not re-evaluated according to the ISUP 2014 recommendations. Biopsy and pathological Gleason score were transformed to GG models. However, in the vast majority of studies, GG was created in the same manner [10]. The retrospective nature of the study might introduce potential selection bias, a relatively low number of final events (i.e., cancer-related death), and more than 15% missing data could have an impact on the final results and reduce generalizability of our findings. Finally, we did not have a possibility to compare our findings with outcomes after radiotherapy, which is another radical treatment option for localized PCa.

However, we should notice some strengths of the presented analyses: long-term follow-up, competing risk analysis, corrected data of cause of death and in detail presented CSM and OCM using GG model after RP.

## 5. Conclusions

CSM and OCM differed widely between GGs. GG model is an independent predictor of CSM and could be successfully used for interpretation of long-term risk of CSM and OCM following radical prostatectomy. It is also helpful for personalized clinical decision making.

## Figures and Tables

**Table 1:** Descriptive characteristics of 1921 prostate cancer patients treated with radical prostatectomy.

Parameter	All Patients *n* = 1921	Age <65-Year *n* = 1016	Age ≥65-Year *n* = 905	*p*
Age, years—median (IQR)	64 (59–68)	59 (56–62)	68 (67–71)	<0.001
PSA, ng/mL—median (IQR)	6.6 (4.8–10.1)	6.2 (4.6–9.8)	6.9 (51–10.4)	<0.001
Clinical stage (cT), *n* (%)				
T1	522 (27.2)	294 (28.9)	228 (25.2)	
T2	1092 (56.9)	563 (55.4)	529 (58.5)	
T3	304 (15.8)	158 (15.6)	147 (16.2)	0.2
Unknown	2 (0.1)	1 (0.1)	1 (0.1)	
Biopsy Gleason score (ISUP GG), *n* (%)				
1	1099 (57.2)	591 (58.2)	508 (56.1)	
2	583 (30.3)	318 (31.3)	265 (29.3)	
3	88 (4.6)	47 (4.6)	41 (4.5)	0.003
4	99 (5.2)	33 (3.2)	66 (7.3)	
5	52 (2.7)	27 (2.7)	25 (2.8)	
Pathological stage (pT), *n* (%)				
T2	1169 (60.9)	633 (62.3)	536 (59.2)	
T3a	579 (30.1)	299 (29.4)	280 (30.9)	0.3
T3b-4	173 (9.0)	84 (8.3)	89 (9.8)	
Pathological Gleason score, (ISUP GG), *n* (%)				
1	502 (26.1)	268 (26.4)	234 (25.9)	
2	975 (50.8)	534 (52.6)	441 (48.7)	
3	210 (10.9)	125 (12.3)	85 (9.4)	<0.001
4	103 (5.4)	38 (3.7)	65 (7.2)	
5	131 (6.8)	51 (5.0)	80 (8.8)	
Surgical margins status, *n* (%)				
Negative	1234 (64.2)	668 (65.7)	566 (62.5)	
Positive	606 (31.5)	306 (30.2)	300 (33.1)	0.3
Unknown	81(4.2)	42 (4.1)	39 (4.3)	
Lymph nodes status, *n* (%)				
pN0	651 (33.9)	306 (30.1)	345 (38.1)	
pN1	81 (4.2)	37 (3.7)	44 (4.9)	<0.001
Unknown	1189 (61.9)	673 (66.2)	516 (57.0)	
Post RP treatment, *n* (%)				
Adjuvant	63 (3.3)	28 (2.8)	40 (4.4)	0.4
Salvage ADT	75 (3.9)	29 (2.8)	46 (5.1)	0.1
Salvage RT ± ADT	291 (15.1)	128 (12.6)	163 (18.0)	0.08

PSA—prostate specific antigen, GS—Gleason Score, ISUP GG—International Society of Urological Pathology Grade Group, RP—radical prostatectomy, pN0—negative lymph node, pN1—positive lymph node, ADT—androgen deprivation therapy, RT—radiotherapy.

**Table 2:** Multivariable competing risk analysis of cancer-specific mortality.

Cancer Specific Mortality			
Pathological Parameter	HR	95% CI	*p*
Preoperative PSA (ng/mL)	0.99	0.95–1.02	0.4
Age (year)	1.1	1.03–1.14	0.001
Pathological stage			
pT2		Referent	
pT3a	1.02	0.43–2.41	0.9
pT3b-4	3.1	1.28–7.49	0.01
Pathological ISUP GG			
GG 1		Referent	
GG 2	1.9	0.58–6.02	0.3
GG 3	4.7	1.18–18.47	0.03
GG 4	6.8	1.87–24.76	0.004
GG 5	20.3	5.55–74.31	<0.0001
SM status			
Negative		Referent	
Positive	2.3	1.16–4.64	0.02
Unknown	2.2	0.47–9.91	0.3
LN status			
Negative		Referent	
Positive	3.2	1.47–6.88	0.003
Unknown	0.8	0.38–1.59	0.5

HR—hazard ratio, CI—confidence interval, PSA—prostate specific antigen, ISUP GG—International Society of Urological Pathology Grade Group, SM—surgical margins, LN—lymph nodes.

**Table 3:** Cumulative 5- and 10-year mortality from cancer and other causes in all cohort and subgroups stratified according to age.

ISUP GG	5-Year Mortality (95% CI)		10-Year Mortality (95% CI)	
	Prostate Cancer	Other Causes	Prostate Cancer	Other Causes
Pathological ISUP GG, all patients				
1	0.2 (0.05–0.4)	4.5 (3.7–6.1)	0.9 (0.3–2.2)	15.5 (11.8–18.4)
2	0.4 (0.2–0.8)	4.8 (3.9–6.3)	2.3 (1.3–3.9)	16.1 (12.8–18.7)
3	1.4 (0.6–3.0)	4.4 (2.4–7.7)	7.6 (3.9–14.8)	12.6 (7.9–22.9)
4	2.8 (1.1–7.1)	7.1 (4.0–11.2)	14.7 (7.3–29.3)	17.7 (12.6–32.7)
5	11.3 (7.2–17.6)	7.9 (4.1–12.1)	48.6 (33.9–69.7)	6.5 (2.6–21.2)
Pathological ISUP GG (<65-year)				
1	0.1 (0.0–1.2)	3.8 (2.0–4.8)	0.4 (0.0–3.0)	9.4 (5.9–14.2)
2	0.1 (0.0–0.4)	4.7 (2.6–5.5)	0.3 (0.0–2.1)	11.3 (7.9–14.9)
3	1.8 (0.6–5.3)	4.0 (1.1–7.9)	7.3 (2.1–25.7)	7.0 (3.9–18.8)
4	1.9 (0.2–14.3)	5.3 (1.0–12.5)	7.6 (1.4–41.9)	9.8 (3.4–31.7)
5	15.9 (6.8–37.2)	6.1 (0.9–15.3)	50.7 (27.6–93.3)	0.0 (0.0–38.9)
Pathological ISUP GG (≥65-year)				
1	0.3 (0.0–0.8)	6.3 (4.7–9.3)	1.4 (0.5–4.2)	22.1 (16.7–26.8)
2	0.8 (0.4–1.6)	6.2 (4.8–8.4)	4.3 (2.4–7.6)	20.6 (16.8–24.7)
3	1.5 (0.4–5.6)	5.9 (3.0–11.4)	8.1 (2.8–23.4)	18.1 (10.1–35.5)
4	3.7 (1.5–9.5)	8.9 (4.6–16.6)	18.9 (9.9–36.1)	22.6 (16.9–43.3)
5	10.7 (6.0–19.0)	9.7 (4.6–17.9)	46.7 (30.2–72.0)	12.8 (6.8–56.9)

CI—confidence interval, ISUP GG—International Society of Urological Pathology Grade Group.

**Table 4:** Cumulative 10-year overall mortality and cancer-specific and other-cause mortality ratio according to grade group in all cohort and subgroups stratified by age.

ISUP GG	OM (%)	Proportion of CSM (%)	Proportion of OCM (%)	Ratio CSM/OCM
Pathological ISUP GG, all patients				
1	16.4	5.5	94.5	1:17
2	18.4	12.5	87.5	1:7
3	20.2	37.6	62.4	1:2
4	32.4	45.4	54.6	1:1
5	55.1	88.2	11.8	7:1
Pathological ISUP GG (<65-year)				
1	9.8	4.1	95.9	1:23
2	11.6	2.6	97.4	1:37
3	14.3	51	49	1:1
4	17.4	43.7	56.3	1:1
5	50.7	100	0	100:0
Pathological ISUP GG (≥65-year)				
1	23.5	6	94	1:16
2	24.9	17.3	82.7	1:5
3	26.2	30.9	69.1	1:2
4	41.5	45.5	54.5	1:1
5	59.5	78.5	11.5	7:1

OM—overall mortality, CSM—cancer-specific mortality, OCM—other-cause mortality, ISUP GG—International Society of Urological Pathology Grade Group.

## Data Availability

The data presented in this study are available on request from the corresponding author.
